# Towards Breathing as a Sensing Modality in Depth-Based Activity Recognition

**DOI:** 10.3390/s20143884

**Published:** 2020-07-13

**Authors:** Jochen Kempfle, Kristof Van Laerhoven

**Affiliations:** Department of Electrical Engineering and Computer Science, University of Siegen, 57076 Siegen, Germany; jochen.kempfle@uni-siegen.de

**Keywords:** activity recognition features, non-contact respiration estimation, depth imaging

## Abstract

Depth imaging has, through recent technological advances, become ubiquitous as products become smaller, more affordable, and more precise. Depth cameras have also emerged as a promising modality for activity recognition as they allow detection of users’ body joints and postures. Increased resolutions have now enabled a novel use of depth cameras that facilitate more fine-grained activity descriptors: The remote detection of a person’s breathing by picking up the small distance changes from the user’s chest over time. We propose in this work a novel method to model chest elevation to robustly monitor a user’s respiration, whenever users are sitting or standing, and facing the camera. The method is robust to users occasionally blocking their torso region and is able to provide meaningful breathing features to allow classification in activity recognition tasks. We illustrate that with this method, with specific activities such as paced-breathing meditating, performing breathing exercises, or post-exercise recovery, our model delivers a breathing accuracy that matches that of a commercial respiration chest monitor belt. Results show that the breathing rate can be detected with our method at an accuracy of 92 to 97% from a distance of two metres, outperforming state-of-the-art depth imagining methods especially for non-sedentary persons, and allowing separation of activities in respiration-derived features space.

## 1. Introduction

Respiration is a vital function of our human body, bringing in oxygen as we inhale and sending out carbon dioxide as we breathe out. The rate of respiration is one of the most important vital signs [1]. It usually lies between 12 to 16 breaths per minute, and tends to change with physical exercise, fever, illness, and with a range of conditions [2]. Monitoring and guiding a subject’s respiration has a wide range of applications that coincide with activity recognition applications, including medical diagnosis and treatment, sleep assessment, sports and fitness, or mindfulness and meditation exercises.

We argue in this article that breathing as a modality for activity recognition can complement well that of user body posture tracking, which is often central in vision-based action recognition from the user’s environment. For instance in sports and fitness applications, it is required to learn and maintain a specific breathing technique, while the respiratory rate is an important indicator of an athlete’s performance. The importance of tracking the respiratory rate and the already existing variety of contact-based measurement methods for instance is summarized in [3]. Depth cameras, although being less obstructive, remain underrepresented in this domain due to their susceptibility to physical movements. They can however play an important role once such limitations are removed, and can help in distinguishing breathing-specific activities and provide user posture using the same modality.

In human activity recognition, vital signs such as heart rate and respiratory rate have been shown to be of importance to improve recognition accuracy [4] by for example helping to distinguish between activities with otherwise similar motion features [5]. Respiration may also give valuable insights into the quality of an executed activity, for example during sports exercises or meditation. Furthermore, information about a user’s mood or drowsiness, both being reflected in breathing, may further be interesting features in some scenarios such as driving a car.

Many sensors have been proposed and implemented to detect a user’s respiratory rate, including respiration belts worn around the chest, mask-like spirometers, skin-based photoplethysmography, or body-worn inertial sensors, such as in [6]. Most methods require physical contact to the user’s body, which tends to become burdensome, uncomfortable, or restraining for the person to wear such sensors over longer time periods. Especially for users that perform breathing-related activities such as meditation or paced-breathing exercises, body-worn devices tend to cause distraction and require an extra step in the setup. It is notoriously difficult to obtain a respiration signal from a distance, though. Recent studies have demonstrated that users’ respiration rates can be obtained from depth camera images in certain, well-defined scenarios. The assumptions and conditions in which such prior methods were evaluated, however, have remained limited and far from realistic: Cameras had a direct line of sight to the user’s torso, and users cannot perform activities other than lying down or sitting still.

**Figure 1:**
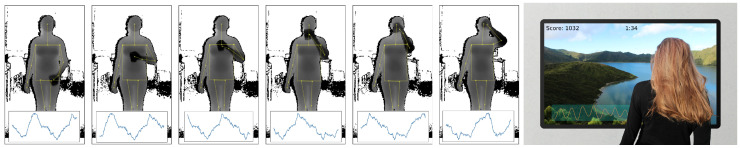
**Left:** Our approach estimates the breathing signal and rate of a user, using body posture data and the raw depth signals from a depth camera in the environment. The user is assumed to be indoors and facing the camera, for instance while performing activities guided by a display, but can be anywhere in the frame, upright or sitting, and she can perform activities that self-occlude the torso. **Right:** An example application with the user performing a relaxation exercise with guided respiration while her breathing is tracked and evaluated over time.

This paper presents a novel approach to monitor a user’s respiration from a depth camera that the user is facing, which does not require the user to sit or stay perfectly still or to be at a predetermined position or distance to the camera, and is robust to users occasionally blocking their upper bodies (as illustrated in Figure 1). As many RGB-D approaches for activity recognition rely on extracting body pose (and sequences thereof), respiration features could thus be seen as an additional modality to specify certain activities. Examples could be the quality of breathing for weightlifting exercises, the regularity of breathing for meditation, or the breathing speed reflecting affect or drowsiness while driving car or watching TV. We argue that respiration as an additional modality can enhance the field of activity recognition in certain tasks, as it provides feedback on a user’s condition, such as state of health, effort, affect, or drowsiness, and enables direct bio-feedback for these.

We present in this paper a novel method and its crucial parameters for estimating a user’s breathing from a distance using depth camera data, to enable respiration as supplementary feature in depth-based activity recognition scenarios, without any additional hardware requirements while being non-intrusive and computationally efficient. Our method is designed for dynamic situations, where the user can be (1) anywhere in the camera’s frame, (2) sitting or standing, leading to additional motion that can be expected to interfere with respiration rate estimation, and (3) have their upper bodies occasionally occluded. Our contributions are threefold:

- A novel method is proposed that is able to perform respiration signal and rate estimation under several challenging conditions including user motion and partial occlusions of the user’s chest. This method works under the assumptions that the observed users are indoors and generally facing a depth camera, and relies on a model of the user’s torso that detects and corrects for occlusions, time domain noise filtering, and an estimation of breathing by observing differences in an adaptive, maximally relevant window of depth pixels.
- A series of experiments on this dataset is presented, in which we validate our method against ground truth from a respiration belt, compare it to related approaches, and highlight the used parameters and design choices for our approach which deliver the best results. Results show that especially for the standing upright and occlusion situations, our method shows a more robust and accurate estimation of breathing rate, compared to previous methods.
- On this dataset, we show that the obtained respiration signal from our method can be used to generate breathing-related features that characterize and separate several breathing-specific activities that would otherwise, for instance by observing the user’s body pose, be hard to detect.

## 2. Related Work

Systems that are able to monitor a user’s breathing have been presented in the past for several applications and scenarios, with many health care and fitness-related aspects as a main motivation. This section, after motivating the use of breathing as a modality for activity recognition based on recent research, gives an overview of previous work in respiration detection, focusing in particular on non-invasive methods, as well as optical and depth-sensing methods.

### 2.1. Breathing as a Modality for Activity Recognition

Only a few studies in the domain of human activity recognition have thus far incorporated respiration into their experiments. Centinela [7] uses acceleration data in combination with vital signs, including respiratory rate, to distinguish different activities, namely walking, running, sitting, ascending, and descending. It is reported that vital signs together with acceleration data can be useful for recognizing certain human activities more accurately than by considering acceleration data only, especially in the case when acceleration signals are similar. The classification of some activities on the other hand did not benefit from the additional vital sign data. In [5], physiological data, including respiratory rate, obtained from a wearable sensing device is used as auxiliary modality to discriminate between four activity classes, namely lie, sit, walk and jog. To recognize lifestyle activities of diabetic patients [8], WiFi, GPS, sound and acceleration data from a smartphone, as well as heart rate and respiratory rate from a ECG monitor are used to distinguish ten different classes. The effects of respiratory rate on classification accuracy for the latter two studies however was not evaluated.

An interesting application is Go-with-the-Flow [9], where body and movement awareness is enhanced through sound feedback to rebuild confidence in physical activity for patients with chronic pain. The sound feedback is generated based on a patient’s movement and breathing. The patient’s posture, inter alia, is tracked via a Kinect sensor whereas breathing is assessed with two wearable respiration sensors. Because breathing rate rises with anxiety, and patients often hold their breath if they are anxious or overly focused on a movement, the system, based on evaluating the respiration data, produces sound signals as a prompt to breathe calmly.

Recent research shows how a subject’s respiration signal can be used for drowsiness detection while driving a car [10,11]. Also emotion classification from respiration and other physiological features has been a focus in some studies [12,13]. In [13], an accuracy of emotion classification of 75 to 90% from breathing alone is reported, depending on the chosen feature within the respiration signal. Finally, there exists a dataset of functional senior fitness tests [14] that comprises acceleration readings at the hip, posture data from the Kinect, and ECG, respiratory rate and blood volume pressure from physiological sensors. The dataset is meant to develop algorithms to automate the assessment of fitness levels.

In many of these studies, either a depth sensor already is used to track a person’s movements, or a depth camera could easily be deployed, enabling a remote sensing of a person’s breathing and effectively reducing the amount of sensors to wear. The following sections will give an overview of previous work on measuring respiration from a distance, especially with a focus on depth sensors.

### 2.2. Remote Respiration Estimation

Respiration estimation from standard RGB and near-infrared cameras has been proposed early on. Methods employed in this area range from image subtraction-based techniques such as the proposed method of Tan et al. [15], to optical flow-based methods as presented by Nakajima et al. [16] and Kuo et al. [17]. In Bauer et al. [18], the respiration is measured by combining optical flow methods with data from a depth sensor and it is reported that the respiration measurement based on optical flow delivers a more accurate respiratory rate estimate compared to mere Time of Flight (ToF) depth measurements.

More recently, several works have focused on the detection of breathing rate and non-invasive detection of breathing-related disorders with RF monitoring systems that extract the breathing signal from the wireless channel. UbiBreathe [19] for instance presents an approach that works on WiFi-enabled devices, even when not held by the user. Evaluations on three study participants have shown that under certain settings such an approach works well, but is heavily influenced by user’s motion and on the location of the wireless access point and the wireless device. Furthermore, the TensorBeat system [20] employed CSI phase difference data to obtain the periodic signals from the movements of multiple breathing chests by leveraging tensor decomposition. Their work shows in a larger-scale experiment in multiple environments that breathing rate estimation is particularly challenging as more people are present in the environment. In [21], with the Fresnel model, the underlying physical principle for RF-based respiration monitoring is derived. It is shown how WLAN-based respiratory rate detection depends on location and orientation towards receiver and transmitter, and how a two user respiratory rate detection under ideal conditions is challenging. Both users need to breath at a different pace to be able to distinguish the signals and it is not possible to assign a signal to the respective person. The location dependency was leveraged by [22] through conjugate multiplication of CSI between two antennas.

Depth-based respiration measurements rely on the observation that during respiratory cycles, the change of the volume of the person’s chest and abdomen can be spotted by a standard depth sensor as inhalation and exhalation will bring these regions in closer or larger distance to the depth sensor. These changes in distance typically have a magnitude of approximately 10 mm for normal breathing. When the remainder of the person’s body remains motionless, these readings will directly reflect the inhalation and exhalation of the person.

The majority of previous works capture the respiration related change of distance by averaging the depth pixel values within a certain bounding box aligned to the observed body region, such as the chest. The general idea behind this is that within this region the breathing motion is expected to cause most depth pixels, and thus the average among all pixels, to correlate with the respiratory rate.

While in an early proof of concept [23], a rectangular selection containing the chest of the examined person is hand-annotated, more recent works often use fiducial markers or rely on joint position estimates such as those given by the Kinect framework to define the region of interest for the computation of the mean. In [24], a first evaluation for different parameters in mean-based respiration monitoring is conducted. The parameters are varying sampling rates between 5 Hz, 7 Hz, and 9 Hz, different orientations (0° or 25°), three different light intensities, and variable clothing worn by the observed person (sweater, jacket, and T-Shirt). The evaluation is approach-specific, however, and results show that these few parameters tested have in the end little effect on the proposed algorithm’s performance. In [25] the quality of the respiration signal for a mean-of-depth based method is evaluated under varying parameters, such as distance or bounding box position and size for in total 7 users sitting in front of a Kinect v2. The main findings are that smaller distances, higher sampling rates, and observing the chest region only are best suited for the respiration estimation in terms of signal quality. Centonze et al. [26] and Schätz et al. [27] use a depth camera for the special case of observing the respiratory rate of sleeping persons. By using features that contain the frequency and the regularity of the breathing, the sleep states (being awake, in REM, or non-REM) are classified. The respiration signal is computed from the mean (or the mean of pixel-wise differences over two successive depth frames [27]) of a manually selected region in the depth image that corresponds to the sleeping person’s chest area. With the addition of RGB data that is available in many depth cameras, extra biophysical information can be extracted. Procházka et al. [28] for instance combined the respiratory rate with estimates of the heart rate, measured by using a built-in RGB and infrared camera to detect the slight changes around the mouth caused by blood pressure changes for each heart beat. The respiratory rate is, as in previous work, obtained by computing the average over a rectangular selection of depth pixels at the torso. The respiration signal time series is band-pass-filtered with finite impulse response filtering of order M = 40 with coefficients defined to form a band-pass filter with cut-off frequencies 0.2 Hz and 2.0 Hz and is applied in both forward and reverse directions. The respective cut-off frequencies are set in such a way that the frequency components that are not part of breathing or the heart rate are rejected.

In other works, the volume of the user’s chest or torso explicitly is modelled from depth data. Aoki et al. [29] use the Kinect’s shoulder and hip joint position estimates as boundaries for a rectangular selection and convert the included depth values to 3D coordinates. The volume then is computed by using Delaunay triangulation with linear interpolation. The observed volume is argued to be proportional to the air volume measured by a spirometer and has been called quasi-volume by the authors. The proposed method was evaluated by monitoring 6 male study participants on a bicycle ergometer, pedaling at a constant speed and with the motion artifacts present in the obtained signal. These motion artifacts, due to the known pedaling frequency of about 1 Hz, could subsequently be filtered out with a band-pass filter with a bandwidth of 0.1 Hz to 0.7 Hz. In [30] the respiration signal also is computed by a volume-based approach, but in comparison to a simple mean-based approach, it has been shown to be less accurate while being computationally much more expensive.

Previous work has also suggested the explicit modeling of respiration to obtain more reliable estimates, by using principal component analysis (PCA) over a predefined area of the user’s chest for a certain number of successive depth images. Ref [31] for example uses the PCA of the user’s torso and applies the varimax rotation to acquire the obtained PCA model. The model this way has more relevance to respiration than the model from the standard PCA, which contains more variations. The principal axes obtained from the model were found to feature local deformations that are highly correlated to thoracic and abdominal breathing. By attaching a zoom lens to a Kinect v1 IR projector, ref [32] increase the tracked dots’ size and record the trajectories of each dot. PCA is applied to the resulting matrix and the 16 strongest components are kept. This approach was tested on 9 sleeping study participants which were positioned in an optimal view and distance (2 m) of the depth camera. Ref [33] use white markers that are visible in the RGB data to define the region of interest for the depth data from an Asus Xtion PRO RGB-D camera. A PCA of the first 100 frames is computed to obtain a motion model for the breathing, after which the first three principal components are used to reconstruct noise-free data. The resulting depth values then are used to generate a surface mesh and to compute its volume, which has shown strong correlation to spirometer data in a study on patient-specific models.

Our method assumes in contrast to most of the above approaches that the depth camera is at an unknown distance to the user and that the user’s position is not known beforehand. Additionally, users are not limited to having to lie down or remain sedentary without moving, but instead can be standing upright and move their arms and hands in front of their chest and abdomen area. Before we validate our proposed method for its use in activity recognition, we first detail our proposed method in the next section.

## 3. Method Description

**Figure 2:**
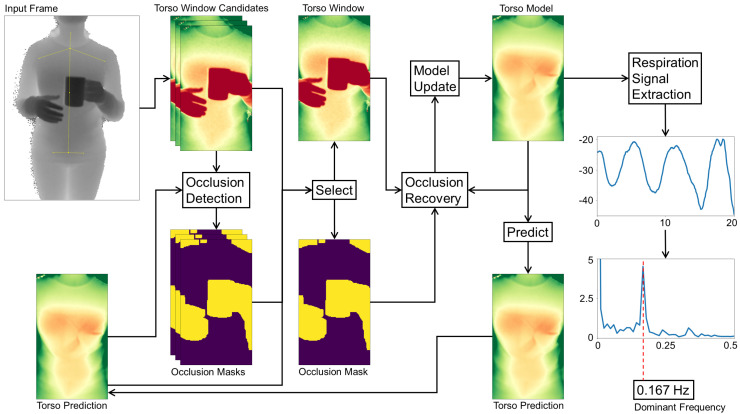
The core steps of our respiration monitoring method. From left to right: The process start with the camera’s depth *input frame* and the estimated torso position of the user. Since joint position estimates contain jitter, multiple *torso window candidates* that frame the torso are selected. Combined with the torso prediction from the previous frame, each candidate is then assigned an occlusion mask. The best matching candidate to the torso prediction is selected as *torso window* and forwarded to the occlusion recovery stage, which uses the occlusion mask as well as the torso prediction from the previous frame to yield the current *torso model*. The torso model then delivers the prediction for the next frame, where it will be used for occlusion recovery. The torso model is transformed to a single respiration state value, the history of these values yields a respiration signal that, after Fourier Analysis and extraction of the *dominant frequency*, estimates the respiratory rate.

This section gives an overview of our proposed method to monitor a user’s respiration from subsequent depth camera frames. We focus on an indoors setting where a user is facing a depth camera, which also tracks the user’s body joints. The data processing chain of our method is divided into different stages that each perform an abstraction from the individual input depth frames to the final respiratory rate estimates. A sketch of the overall process is provided in Figure 2.

Starting from the camera’s single raw depth frames, users and their postures are first identified. For each user, the body’s joint positions are used to mark the user’s torso (Section 3.1), with especially the respiration-related regions of the chest and abdomen being of high interest. With the help of our model we can identify and mask out occluded parts and refine the alignment of the torso window in case of a mismatch of the estimated joint positions. The selected region and its occlusion mask (Section 3.2) subsequently are forwarded to the occlusion recovery stage. Here, all occluding parts from the depth image are replaced by a model-generated approximation of the torso surface, also removing any salt and pepper noise present in the depth image. The individual steps of the occlusion recovery are presented in Section 3.3 and our adaptive model implementation is specified in Section 3.4. The resulting recovered depth image satisfies our criteria of being well aligned and keeping sufficient detail in occluded areas such that it can be used to update the model without losing its integrity. The following stage extracts the respiration signal in a movement-robust fashion by identifying the torso deformation caused by breathing as described in Section 3.5. From the resulting breathing signal, the respiratory rate can finally be estimated through Fourier analysis.

### 3.1. Locating Users and Torso Windows

The incoming depth frames are first scanned for users present in the scene and, if any are detected, the users’ torso areas are tracked over time to extract respiration-relevant motions.

**Input Frame.** Tracking is done by a skeletal model consisting of 25 joints, revealing the person’s position and pose, following the approach of [34]. We use the implementation in the Kinect SDK 2.0, which is capable of tracking up to six persons simultaneously. Knowing a user’s body posture enables us to mark the *torso window*, which reveals slight motion across the torso surface during respiratory cycles. The decisive anchor points to determine the torso position and scale horizontally are the left and right shoulder joints. In the vertical direction, the neck, the shoulder mid, the spine mid, and hip joints are used. The estimated joint positions can be expected to be slightly unstable over time, jumping between neighbouring pixel positions. In some cases, in the presence of occlusions or arm movement for instance, the joints may also be misaligned. Especially when users are further away, such jumps or misalignment occurrences are more likely.

**Torso Prediction.** The depth input frames typically comprise several challenges, including noise, different scales and extents of the user’s torso region, background and outlier pixels, occlusions and shadowing, motion artifacts, and surface deformations such as folds caused by clothing. To overcome these challenges, we maintain an adaptive model for each user, which estimates the torso window appearance over time. More details on how this model is constructed and updated will be given in Section 3.4.

**Torso Window Candidates.** Our approach contains some heuristics to stabilize the tracking of the torso window. First, the window’s aspect ratio is fixed to a predefined value at initialization. The torso window’s change in size caused by small swaying movements while standing is negligible, so a fixed height and width are assumed across frames. To cater for users moving strongly towards or away from the depth camera, several rescale operations would need to be performed to fit the window in the next step. Second, the position of the torso window, although allowed to move in the frame, is clamped to a position with similar content of the predicted depth image from our model. For this, all possible $x_{win}$ and $y_{win}$ positions that fall in between the current and previous window position are permuted to form as many different *candidate windows* for the torso. All candidates obtained from $I(x_{win}+x,y_{win}+y)$ are compared pixel-wise to the model prediction image $I_{predict}\left(x,y\right)$ as defined in (1):

(1) $$\begin{array}{c} I_{best\;fit}=\underset{x_{win},y_{win}}{arg\;min}\sum_{x,y\in N}^{w,h}M_{win}^{-1}\left(x,y\right)\cdot {\left(I\left(x_{win}+x,y_{win}+y\right)-I_{predict}\left(x,y\right)\right)}^{2} \end{array}$$

By multiplying the energy term with an inverted occlusion mask $M_{win}^{-1}\left(x,y\right)$ of the respective candidate window win, all detected occlusion pixels are ignored, as they unnecessarily increase the difference of both images, pushing the window away from the occlusion instead of matching the surface. The best matching window is selected as the torso window and forwarded to the Occlusion Recovery step. The next section will first detail the procedure of selecting an occlusion mask.

### 3.2. Occlusion Mask

The presence of occlusions poses a particularly hard problem for optical respiration monitoring, as (1) important regions of the torso may be blocked, and (2) movement of the occluding entity may be misinterpreted as evidence for a respiration signal. It is, therefore, important to detect and mask any entities that occlude the torso with an occlusion mask *M*. This mask helps maintaining a breathing-relevant set of depth pixels and ensures the integrity of our model. To identify and mask occlusions, knowledge about the user torso’s surface in the current frame is required. Our model predicts the surface appearance and distance of the tracked torso. Since occluding entities will be in front of the torso, their corresponding depth values will be smaller. The difference matrix obtained from subtracting the model prediction from the corresponding input frame therefore yields negative values for occlusions. To find the occlusion mask, each pixel in the difference image is compared to a certain threshold that defines the minimum distance to the torso surface. Anything above this threshold is considered part of the user torso surface, including skin deformations or clothing. Objects that do erroneously find their way into the model will be excluded from the model as soon as the blocked torso surface becomes visible again. The occlusion threshold has to take into account that the model has a small delay due to the model’s low-pass filtering behaviour, where fast movement may lead to incorrect masking. The same applies to noise in the input frame. In initial studies we found an optimal value for this threshold of 30 mm. Equation (2) mathematically defines the occlusion mask M(x,y) at pixel positions *x* and *y* with the input image I(x,y) and the torso prediction $I_{predict}\left(x,y\right)$ as well as the threshold value $z_{threshold}$:

(2) $$\begin{array}{c} M\left(x,y\right)=\left\{\begin{array}{c} 1\;\;if\;I\left(x,y\right)-I_{predict}\left(x,y\right)<z_{threshold}\\ 0\;\;else \end{array}\right. \end{array}$$

Occluding entities in depth imaging often show a halo on their edges, caused by interference from the emitted infrared light being reflected from the object upon the torso surface. To avoid such a halo leading to undesirable effects in the next steps, the mask is enlarged by a margin of several pixels. With this mask, occluded entities can be removed in the torso window as well as in the torso model.

### 3.3. Occlusion Recovery

With the occlusion mask in place, we can identify occluded pixels in the input frame and, after fitting the candidate windows for the torso window, our model supplies the depth information for the occluded area. After removing all occlusion pixels from the input frame, the remaining depth pixels are fed into the model update routine to yield a partly updated model state with a hole at occluded regions. From the input frame alone it is not clear how to recover the torso surface, so in a first step we use normalized convolution [35] with a Gaussian kernel to in-paint the unknown area. The previous model state, again using the occlusion mask, is separated in two cuts: the model cut describing the valid torso regions, and the model patch describing the occluded torso regions. The hole in the model cut gets in-painted with exactly the same method and parameters as the partly updated model state was filled before. Subtracting the model patch from the in-painted model cut yields a difference patch that describes the torso surface details. This difference patch now in the final step is added to the partly updated model to recover the surface details of the occluded parts. The first and second order time derivatives of the model eventually have to be updated by feeding the state estimate in the corresponding equations in (4).

**Figure 3:**
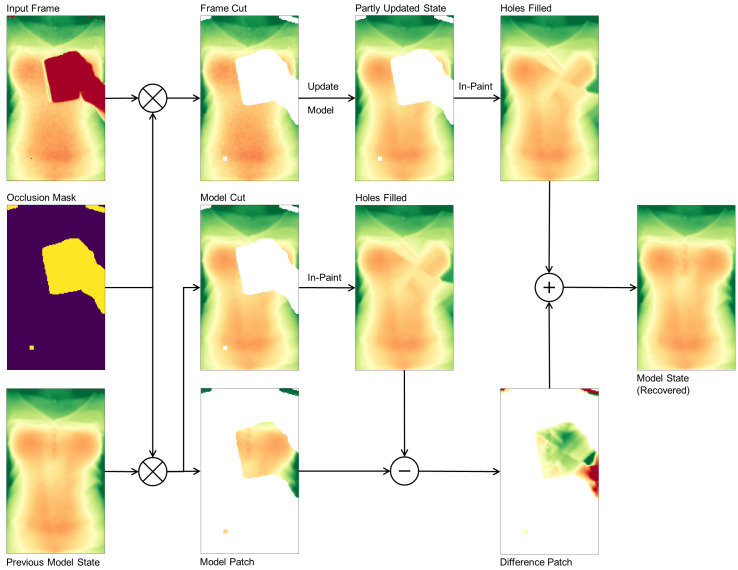
Detailed example of our occlusion recovery process: The depth input frame, occlusion mask, and previous model state are used. The occluded area is removed from the depth frame and the model state, with the model patch from the previous model state kept. The non-zero pixels from the depth input frame cut feed into the model update to yield a partly updated model state. The holes of both model states are in-painted, and the difference of the fitting model patch and the in-painted model cut is added to the in-painted area of the partly updated model state, yielding a new model state. First and second order derivatives are equally updated.

The occlusion recovery plays an important role in our method as it keeps surface details and helps during the occlusion masking of successive frames to not accidentally mask out parts of the torso. The overall process again is depicted in Figure 3.

### 3.4. Adaptive Torso Model

**Figure 4:**
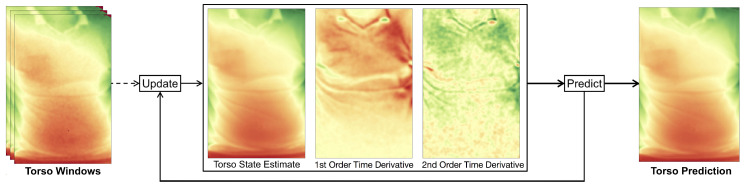
Our approach builds an adaptive model for the user’s torso appearance. From depth input frames, *torso windows* are selected over time that, after occlusion recovery, update the model consisting of a torso state estimate, its first order time derivative, and its second order time derivative. From these, the model can build a torso prediction for the next input frame.

We assume that users are facing the depth camera and thus users’ torso regions will be visible to the depth sensor. Under this assumption, our model becomes a fixed size depth image tracking the torso, along with its first and second order time derivatives (Figure 4). The model parameters are updated at each time step when a new depth frame arrives. With the help of the model, we are thus able to predict the next frame after a single time step by applying (3) to each pixel $x_{t}$ at time *t* independently:

(3) $$x_{t}=x_{t-1}+{\dot{x}}_{t-1}+0.5\cdot {\ddot{x}}_{t-1}$$

#### 3.4.1. Initialization of the Torso Model

To initialize the model, a bounding box around the user torso has to be selected in the input frame. We use the detected body landmarks and joint positions of the person to locate the torso (detailed in Section 3.1). The pixels in the model’s current state estimate are set to the depth values of the selected region in the very first depth frame. This may contain zero valued depth pixels, so-called holes, which are caused by shadowing, occasional salt and pepper noise due to defect pixels, or reflections from certain materials. For the initialization, these holes are filled with the use of a median filter, whereas afterwards, our model will fill the holes during the occlusion recovery stage as described in Section 3.3. The model’s first and second order derivatives are initially set to zero and converge after several frames. The model size, defined by the width and height, is chosen sufficiently large to be able to contain the user’s torso. Since multiple rescaling operations need to be performed if the size of the model is allowed to change, we simplify this in our approach by assuming that a person’s movement perpendicular to the camera is relatively small.

#### 3.4.2. Model Update with Time Domain Filtering

Each depth pixel comprises a certain noise level that increases with distance and follows a Gaussian distribution over time in a stationary scene. In a dynamic scene, however, several objects are allowed to freely move along all axes, so simply taking the mean does not yield an optimal approximation of the scene. As we are only interested in the depth measurements of the user’s torso, we track a bounding box over its x and y position as stated in Section 3.1. Any x and y offsets thus are mapped to approximately the same pixel coordinates of the tracking bounding box. Consequently, only the motion along the remaining z or depth axis remains, and noise can effectively be reduced by a low-pass filter.

For this filter, we use a recursive implementation with a small overhead, in order to achieve a real-time performance. Furthermore, the filter needs to follow the signal closely, i.e., with a small delay with little overshoot or damping, as the resulting filtered model is used to detect potential occlusions. An exponential filter implementation (4) is used, which reacts faster to an input signal than a usual double exponential filter, while having less overshoot. This behavior is achieved by incorporating the first order time derivative ${\dot{x}}_{t-1}$ as well as the second order time derivative ${\ddot{x}}_{t-1}$ in the prediction of the state update $x_{t}$ from the measurement $x_{t,meas}$ at frame *t*. The computation of the second order time derivative ${\ddot{x}}_{t}$ incorporates the difference of the previous and current velocity approximation $\Delta {\dot{x}}_{t}$ as well as a damping factor *d* to reduce the typical overshoot of the double exponential filter. The filtering equations (4) are applied to each pixel separately to yield a smooth state estimate over time and recursively compute our model:

(4) $$\begin{array}{c} x_{t}=\alpha \cdot \left(x_{t-1}+{\dot{x}}_{t-1}+0.5\cdot {\ddot{x}}_{t-1}\right)+\left(1-\alpha \right)\cdot \left(x_{i,meas}\right)\\ {\dot{x}}_{t}=\beta \cdot \left({\dot{x}}_{t-1}\right)+\left(1-\beta \right)\cdot \left(x_{t}-x_{t-1}\right)\\ {\ddot{x}}_{t}=\gamma \cdot \left({\ddot{x}}_{t-1}\right)+\left(1-\gamma \right)\cdot \left(\underset{\Delta {\dot{x}}_{t}}{\underbrace{\left(x_{t}-x_{t-1}\right)-{\dot{x}}_{t-1}}}-\underset{damping}{\underbrace{d\cdot {\dot{x}}_{t-1}}}\right) \end{array}$$

### 3.5. Extraction of Respiration Signal

Once the torso window is defined, several approaches to extract a respiration signal from the resulting depth data have been suggested. Simple yet very effective approaches take for each depth frame the mean of a predefined torso region (for example a window of the chest or a part thereof), and thus track the movement of the chest plane towards and away from the depth camera. This tends to work well for all torso regions that show sufficient respiration motions. It does require the user to keep still, however, as small movements in the range of a few centimeters or even below will affect the measurement. While lying down or sitting on a chair with sufficient back support, this has proven a straightforward easy task. If the user is standing, though, the torso tends to be subject to a much larger amount of movement. This section proposes a new method that relies on observing both (1) torso regions within the torso window that correlate with breathing motion and (2) torso regions that are barely affected by breathing motion.

Determining the body movement that is not interfered by the breathing motion is not an easy task. The limbs, although not being affected heavily by respiration, do not represent the body movement due to their capability of independent movements. The torso, on the other hand, is heavily affected by respiration. This ranges from the up and down movement of the shoulders, the expansion and contraction of chest and abdomen, down to motion at the hip e.g. caused by a belt that moves during abdominal breathing. Furthermore, the clothing plays an important role. A loose dress, for instance, could be hanging from the chest covering the abdomen, leading to breathing movement across the entire torso. Clothing in general leads to varying surface deformations during breathing or movement and it is hard to predict the exact source of the deformation of a garment. These findings render almost all torso regions unsuitable for detecting the body movement as they either are affected by respiration or comprise unpredictable or unrelated motion due to surface deformation.

**Figure 5:**
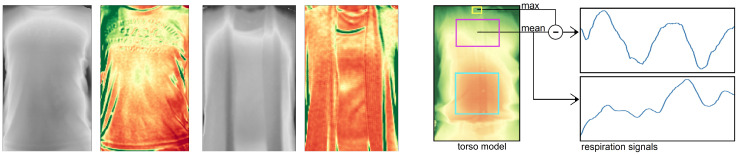
**Left**: Torso surface (left images) and variance of a 12 s time window (right images) of two persons. Red: Low variance; Green: high variance. The throat area shows low variance while the remaining area highly is influenced by clothing. **Right**: The estimation of the respiration signal uses defined areas in the torso window. For the chest region, the result (**top right**) is a clean respiration signal when subtracting the maximum depth measure for a small region around the throat and the mean depth measurement over the chest region. In contrast, the mean depth measurements over the chest alone are highly influenced by motion artifacts (**bottom right**).

In Figure 5 (left) we visualized the variance of the torso from two persons over the course of 12 s with negligible body movements. Low variances are depicted in red and high variances in green. In preliminary experiments, the throat area was found to be a relatively stable region that barely shows movement caused by chest expansion during breathing. In indoor environments it furthermore tends to be left uncovered from a scarf or a tight jacket. The throat however may partly be occluded by a collar which causes, due to breathing motion, a significantly higher variance than the throat. By only considering the furthest points from the depth camera at a certain region around the throat, the effects of the closer points of a moving collar can be minimized. We chose the 90th percentile of that region to become a measure of the body movement.

By combining the above observations, our approach delivers a motion-robust respiration signal by taking the difference of the mean of a highly breathing-affected area, such as the chest, abdomen, or the entire torso, and the maximum value of a minimally affected area, for which the throat is selected as a good candidate. The affected area is defined with a margin of 20% the window size to the left and right (see [25] for the benefits of a slightly smaller window) and the according vertical position and extent as given by the joints as shown in Figure 5 right. The respiration signal is extracted from the torso model, including regions that currently are subject to occlusion recovery. Simply leaving these regions out, due to the torso’s uneven surface structure, would lead to significant signal distortions or, in case of full occlusion of the window, yield no signal at all.

## 4. Study Design and Overview

To validate our system under realistic circumstances, we performed three studies to assess (1) how well our method compares to the actual respiration signal from a chest-worn respiration sensor, (2) how different activities such as sitting, standing, and drinking play a critical role in our method and which of these work particularly well, and (3) which features from the resulting respiratory signal would work well in activity recognition.

### 4.1. Setup and Environment

We ask the participants in the following experiments to position themselves comfortably in front of our sensor setup. This setup entails a Kinect v2 RGB-D camera mounted such that it is aligned to the field of view of an auxiliary display and any user faces both the depth sensor and the display. The depth camera was for all recordings fixed to the height of 1.40 m at an angle of 0° and its raw depth frames and body joint estimates were recorded. For validation of our method, we recorded participants in a well-lit indoors environment, though lighting conditions were challenging as the room has two adjacent walls with large windows along the entire length of the walls.

**Comparison with a wearable respiration belt.** For the comparison of our approach to a method that delivers the chest elevation from a wearable chest strap, a Vernier GDX-RB respiration belt was worn. This wearable device contains a force sensor tied to a strap, which is to be worn around the person’s chest. The measured force is a proportional measure for the chest expansion during breathing and thus is well suited for the verification of our method. It samples at a rate of 20 Hz and transmits the data to a PC workstation via Bluetooth LE which is synchronized with the depth frames.

**Figure 6:**
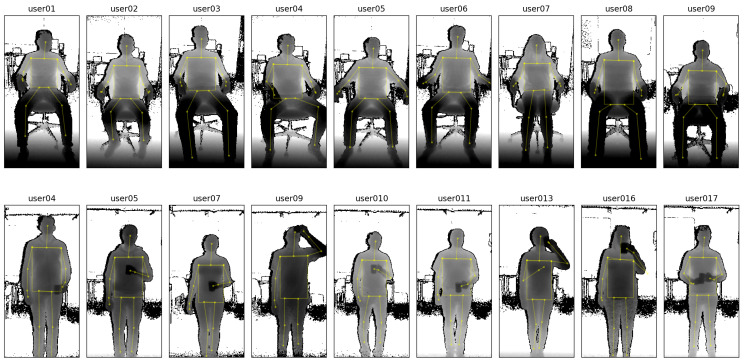
In our evaluations, 24 study participants (of which 7 female, aged between 22 and 57 years old) were asked to sit or stand in front of a depth sensor and were recorded for different conditions. **Top row**: Exemplary depth data from some participants while sitting. **Bottom row**: Some examples of participants while holding a cup (leading to regular occlusions of the torso). All of the above examples are taken from 2 metres distance sessions.

**Study on the influence of user activity.** The goal of our second evaluation study is to validate our method under the variable condition of different user postures and activity. To minimize distractions, participants did in this study not wear the respiration belt, but instead were shown a paced-breathing visualization to ensure that they kept to a fixed respiration rate. Figure 6 shows some examples of the depth data from a distance of 2 m for all participants while sitting, along with some examples while standing upright with occlusions (holding a cup in front of the torso and performing drinking gestures). For the sessions while sitting, the depth camera was positioned with an angle of 25° towards the floor, and for the remaining sessions at an angle of 0°, so that participants were optimally centered in the depth frames.

### 4.2. Study Participants and Protocol

For validation of our method, we recruited 24 participants, 17 of them male. Out of these, 14 (10 male, 4 female) volunteered for the comparison evaluation, and 19 (12 male, 7 female) took part in the study on the influence of user activity. Each participant was beforehand briefed on the study goals and the research questions. They were shown an example of the depth imaging equipment, along with the real-time estimation of respiratory rate. Participants were recruited locally, were not diagnosed with respiratory illnesses, and were instructed to not change their regular indoors clothing, wearing anything from T-shirts, sleeved shirts, sweatshirts and woollen pullovers.

**Comparison study with a wearable respiration belt.** The first evaluation study is conducted with a set of parameters that are particularly challenging for the extraction of the respiratory rate. Recording lasted at least 120 s, with one at a paced breathing rate, and the two others at natural breathing rates. The 14 study participants were instructed to stand in front of the depth sensor and display setup in an upright position at a distance of 3 m while wearing the respiration belt. This setting is challenging to our method due to the distance and due to the standing position that introduces motion artifacts as described in the influence of user activity study’s setting (see Section 6). The belt was worn directly under the armpits, following the instructions in the user manual. Furthermore, the belt’s sensing device due to its form factor is aligned to the side of the body below the left arm and is worn underneath the clothing to not interfere with the measurements of the depth camera. In total three different recordings are captured, each comprising a different activity and breathing pattern:

- A *paced-breathing meditating* recording, where participants were shown a paced breathing display of a growing and shrinking circle with instructions to breathe in and out at the relatively fast frequency of 0.25 Hz (or 15 breaths per minute). This is a common target breathing rate for meditation, whereas a higher-paced breathing experiment would come with its own risks for the study participants.
- A *relaxing* recording, while the display showing a video of landscapes with relaxing background music, to entice a person-dependent slow breathing rate during a relaxing activity.
- A *post-exercise recovering* recording, where participants were monitored after strenuous exercise comprising running down and up 12 flights of stairs, and the display showing the aforementioned relaxation videos. This exercise was chosen to heighten the respiration rate of the study participants, for the safety of the study participants. This recording comprises a high variance of the respiratory rate and, especially in the beginning, many random movements from the user breathing heavily, and is considered even more challenging to our method.

Furthermore, participants were asked to refrain from moving their arms a lot, as the tightening of the breast muscles is known for some users to introduce motion artifacts in the sensor data from the respiration belt.

**Study on the influence of user activity.** In this study, the 19 participants were told to sit through 20 recording sessions for about 5 min each for several parameters, interspersed with short 5 m breaks:

1. In a first condition, participants were asked to sit in an adjustable office chair in front of the depth sensor. The height of the chair was fixed to 0.5 m, but its back support could be reclined and did not need to be used (i.e., participants could lean back or not, as they preferred). To fix the distances between chair and depth camera, markers were taped to the floor to define the exact positions where the chair had to be placed. Participant were asked to face the depth camera and to keep the arms away from the chest area (e.g., on the chair’s armrests) such that the participant’s upper body was fully visible to the depth sensor.
2. In a second condition, the participants were instructed to stand in an upright position following the same rule as in the first session, i.e., to keep their arms away from the torso region. The goal of this session is to observe the torso’s motion while the observed person is standing relatively still, but does not have the support of a chair’s seating and back surfaces. Having to stand upright for several minutes tends to introduce a range of motions that are unrelated to the breathing movements of the torso region; Some participants did move their arms in different positions during the recordings (for instance, switching between hands on the back and hands in the pockets) or repositioned themselves to a more comfortable posture, making it potentially challenging to extract a respiration signal from these data.
3. A third condition introduced frequent occlusions by instructing the participants to hold a cup of tea in front of their torso while standing upright. At the start of the session, participants were recorded for 20 s while holding their cup away from the torso. For the remainder of the session, participants were instructed to occlude their stomach and chest regions with the cup. Such self-occlusions also occur when gesticulating, but the drinking gestures were found to be particularly challenging due to their relatively slower speeds of execution and the larger, additional occlusion of an in-hand object.

These activities were recorded for each participant at a distance of 2 m between participant and depth camera, at two respiration rates, 0.17 Hz and 0.25 Hz. These rates were obtained through paced breathing, in which participants were asked to adhere to a paced visualization shown on the display, making the recordings independent of user specific breathing behaviours and more comparable with respect to the different parameters. The recording was started after about two minutes, to give the respective participant a chance to adapt his or her respiration rate to the given target frequency.

### 4.3. Performance Measures

The accuracy of the different methods’ respiratory rate estimates is obtained from comparing the dominant frequency of the respective signal to that of a given reference signal. The respiration signals are shifted to frequency domain by a Fast Fourier Transform (FFT) with a moving window approach. We choose a window of length *l* and move it over the signal with a step size *s*, splitting the respective signal up in different equally sized segments for the computation of their frequency representation. If their respective peak frequency within the range of 0.1 Hz (6 breaths/minute) to 1.5 Hz (90 breaths/minute) are equal, the signal under the window is considered to be a correct estimate. The number of correctly estimated windows divided by the overall number of windows of a certain parameter set’s signal (e.g., user 1, sitting, 0.25 Hz) is the average accuracy of that parameter set. Due to the frequency binning in frequency domain, the window length also determines the precision.

(5) $$\begin{array}{c} acc\left(x,\omega_{ref}\right)=\left\{\begin{array}{c} 1\;\;if\;\underset{0.1<\frac{\omega }{2\pi }<1.5}{arg\;max}\left(F\left\{x\right\}\left(\omega \right)\right)=\omega_{ref}\\ 0\;\;else \end{array}\right. \end{array}$$

(6) $$Accuracy\left(x\right)=\frac{1}{N}\sum_{i=0}^{N}acc\left(\left[x_{i\cdot s},x_{i\cdot s+l}\right],\omega_{ref}\right)\;\;,\;x=x_{0}\ldots x_{n}$$

As respiration usually comprises a considerably low frequency, a good compromise for the window size is required to test for high accuracy and high precision. A small FFT window size is more accurate in terms of time resolution which means it provides more windows to test the ground truth against, but it cannot resolve a fine frequency resolution, such that a broad spectrum of frequencies will fall into the same frequency bin. A big window size on the other hand may only generate a few test windows for the accuracy computation, and due to their size, they may shadow short occlusion intervals or fail to detect a change in frequency. For example, to yield a frequency resolution with a precision of one breath per minute, a window of length 60 s would be required.

The accuracy measure only tells wether the estimated respiratory rate was within a certain range, but does not give an insight on how far the estimated frequency is off from the ground truth. For this we introduce the accuracy error. It is computed by subtracting the dominant frequencies obtained from our proposed method and the reference signal. The dominant frequencies beforehand are interpolated using Quinn’s second estimator to yield a better frequency resolution as compared to the fixed bin size from the FFT. The error measure this way becomes more precise.

(7) $$Error\left(x,x_{ref}\right)=\left|\underset{0.1<\frac{\omega }{2\pi }<1.5}{arg\;max}\left(F\left\{x\right\}\left(\omega \right)\right)-\underset{0.1<\frac{\omega }{2\pi }<1.5}{arg\;max}\left(F\left\{x_{ref}\right\}\left(\omega \right)\right)\right|$$

The similarity of our method’s estimated breathing time series to a given ground truth signal is assessed by computing their Pearson correlation coefficient (PCC) as given in (8):

(8) $$r_{xy}=\frac{\sum_{i=1}^{n}\left(x_{i}-\bar{x}\right)\left(y_{i}-\bar{y}\right)}{\sqrt{\sum_{i=1}^{n}{\left(x_{i}-\bar{x}\right)}^{2}}\sqrt{\sum_{i=1}^{n}{\left(y_{i}-\bar{y}\right)}^{2}}}$$

Using the Fisher transformation, their respective confidence intervals CI are computed with (9a). The standard error SE, given the sample size n, is computed with (9b), and the z-score z for a desired confidence interval of (1−α)% is obtained from the standard normal cumulative distribution function:

(9a) $$CI=\tanh(\mathrm{arctanh}\left(r_{xy}\pm z_{\alpha /2}\cdot SE\right))$$

(9b) $$SE=\frac{1}{\sqrt{n-3}}$$

Before the computation of the PCC, both signals to be compared are filtered with a 5th-order Butterworth band-pass filter with lower and upper cutoff frequencies of 0.1 Hz (6 bpm) and 1.5 Hz (90 bpm) respectively. This ensures to only compare the relevant frequency bands of respiration, not noise or non-linear offsets over time of both signals under consideration.

The signal-to-noise ratio (SNR) is used as a measure of the signal quality. It is calculated with Equation (10), from which follows that the higher the SNR value, the higher the respiratory signal stands out from the noise and the better it can be extracted from the data.

(10) $$SNR_{dB}=10\cdot \log_{10}\left(\frac{P_{Signal}}{P_{Noise}}\right)$$

## 5. Comparison Study with a Wearable Respiration Belt

Our goal for the first study is to validate our method by comparing its performance to that of a commercial wearable respiration belt. Three different activities were taken as different conditions: following a paced-breathing meditation, relaxing, and recovering after a sports exercise. Before processing, we apply a Butterworth 5th-order band-pass filter to both signals and set the lower and upper cut-off frequencies to the same range we use to find the dominant frequency (see Section 4.3), namely to 0.1 Hz (6 breaths/min) and 1.5 Hz (90 breaths/min). The filter is applied both in forward and backward direction to minimize transients at the start and end of the signal. The band-pass filtering makes the data better comparable for the comparison evaluation as it removes constant offsets and high frequency noise while preserving the breathing-relevant range of frequencies, but is not required in general (see Section 6 for evaluation results without filtering).

### 5.1. Visual Inspection

**Figure 7:**
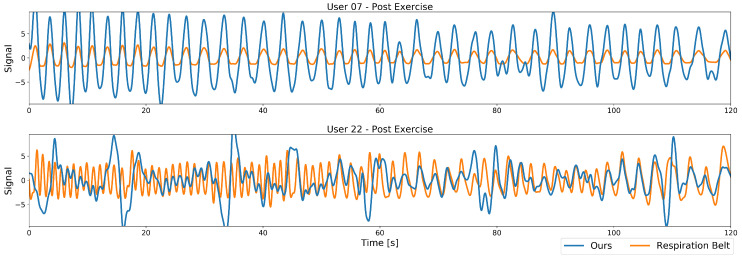
Comparison plots between the output of the chest-worn respiration belt (in orange) and the output of our proposed model (in blue), for the post-exercise condition (going from a fast respiration rate in the beginning to slower one over time). The top segment has a PCC of 0.95 and an accuracy of 100%, both signals match well in terms of frequency. The bottom segment has a PCC of 0.26 and an accuracy of 22%, with the larger peaks on the left in our output due to the user occasionally tilting the torso forward and occluding the throat region with the head.

In a first visual inspection, we examine the differences in the signals of our method. Two example plots of the band-pass filtered respiration signals from our proposed method along with their respective ground truth from the respiration belt are plotted in Figure 7. Both plots are taken after the exercise, resulting in a high dynamic frequency range with a fast respiratory rate at the beginning of the recording (left part of the plots) that decreases over time to a more nominal breathing rate (right part of the plots). Depicted in Figure 7’s top plot are the data from a recording where this works well, with the resulting performance for the Pearson cross correlation of 0.95 and an accuracy of 100%. A second, more challenging recording is plotted below in Figure 7, with a Pearson’s cross correlation of 0.26 and an accuracy of 22%, due to the user moving more during the recording.

At first sight, the main differences lie in signal amplitude and quality. While the best case signal comprises a high amplitude and only small deviations from the ground truth, the worst case signal especially at higher frequencies contains much smaller amplitudes and many peaks and deviations over the whole spectrum. The varying amplitude is caused by the low-pass behaviour of our model, more severe however are the peaks. These stem from the user bending forward while heavily breathing, thus covering his throat with his head. Our method in this case no longer is able to fully reconstruct the relatively small reference region and motion artifacts enter the signal. After such an occlusion event the model is able to recover and the signal follows the ground truth well until the next occlusion occurs. The smaller deviations at lower frequencies are caused by strong movements that could not fully be removed from the signal, but, to a certain extent, still contain the respiration signal.

In general, in terms of frequency, our method matches the ground truth well in both cases, but the many occasional peaks in one signal hinder our method in estimating the correct respiratory rate.

### 5.2. Overall Evaluation Results

**Figure 8:**
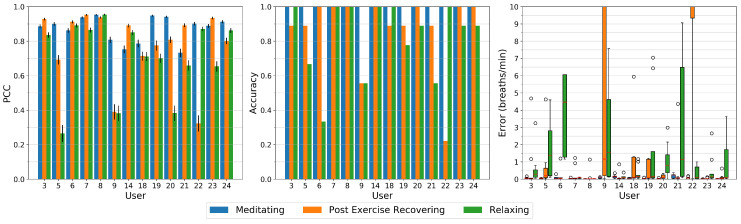
**Left**: The Pearson Correlation Coefficient (PCC) between the data from the Vernier respiration belt and our proposed system, for all users individually. The bars depict the means, while the black bars indicate the 99% confidence intervals. Data from paced breathing (15 breaths per minute) tends to result in high correlation between our system’s prediction and the belt’s output. Relaxed and post-exercise breathing tends to perform slightly worse. **Middle**: The accuracy of our proposed system compared to the respiration belt. **Right**: The error of the estimated respiratory rate compared to the respiratory rate from the respiration belt. Poor performance for users 9 and 22 stem mostly from larger movements during the recording.

After the visual inspection, we quantitatively investigate our methods performance for all study participants and respiration patterns. Figure 8 depicts the Pearson Correlation Coefficient (PCC), the accuracy, and the error of all study sessions. For the accuracy and error measurements, we use a FFT window length of 40 s and a step size of 10 s. To reduce frequency leakage, we apply a Hann-window to those segments.

The high-paced breathing meditation has an accuracy of 100% for all participants and close to zero errors. All signals show high correlation to the respiration belt with a minimum PCC of about 0.75 for users 14 and 21, up to a value of 0.9 and above for about half of the users.

The post exercise respiration shows remarkably high errors for users 9 and 22. All other users either have almost zero errors or errors in the range of about 0.5 to 1.5 breaths per minute as in the case of users 5, 18, and 19. User 9 and 22 also have a significantly lower correlation coefficients below 0.4 as compared to other users, mainly caused by occlusion events where both, due to heavy breathing, lower their head and occlude our algorithms reference region at the throat. In the case of user 22, as described above, the occlusion recovery cannot fully restore that region, leading to peaks in the signal (see Figure 7 bottom). For user 9, this region sometimes can be recovered to a certain extent, but the respiration signal is significantly raised or lowered during that time. These events have similarities in the time domain with a rectangular shape, so lower harmonics show up in the frequency domain that dominate the respiration frequency.

On relaxed breathing, our method performs worse than on the other respiration patterns. Notably some users show a low accuracy with values of 65% for user 5, about 55% for users 9 and 21, and below 35% for user 6. These users also show the highest error rates with a median above one breath per minute, especially for user 6 with a median error of 4.5 breaths per minute. Its PCC however is with a value of 0.9 very high which indicates that due to small signal deformations and due to the small window length used for the low breathing rate, other frequency components become more dominant in frequency domain. Users 5, 9, and 20 have a PCC below 0.4 and can be considered to be only being weakly correlated to the respiration signal from the chest strap. User 20 on the other hand has a high accuracy of about 90% but still a median error of about 0.7 breaths per minute.

**Figure 9:**
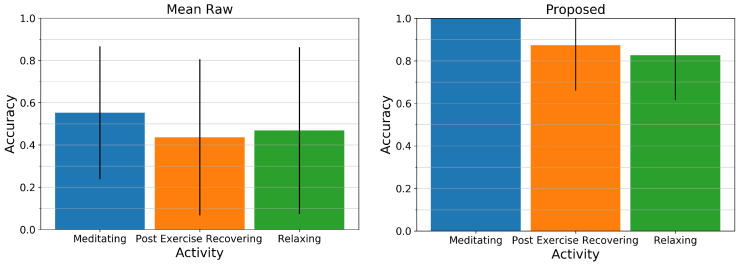
Comparison of two depth-based respiration estimation methods to the respiration belt. **Left**: The mean accuracy and standard deviation of the Mean Raw method. **Right**: The mean accuracy and standard deviation of our proposed system.

Figure 9 displays the mean and standard deviation of the accuracy across all users for the Mean Raw method (left) and our proposed method (right). While the Mean Raw method for all activities only has an average accuracy of about 45 to 55% and a high standard deviation, our proposed method for all activities is above 80% and can estimate the respiratory rate of the paced-breathing meditating exercise perfectly with an accuracy of 100%. The two remaining activities however show relatively large standard deviations as can be retraced in Figure 8 (middle).

To conclude, we observe that for most users and most regular breathing frequencies, our approach performs close to the commercial body-worn respiration belt and closely matches the respiration rate. For the changing frequencies in the post-exercise sessions and the relaxed sessions, a few users did move a lot more during the experiment causing a significant drop in their recordings’ performance. In such cases, such as the one illustrated in Figure 7, multiple breathing cycles are missed and our method under-estimates the breathing rate.

## 6. Study on the Influence of User Activity

The aim of this evaluation is to particularly investigate the influence of user activity in our model, investigating the conditions sitting, standing, or drinking. Contrary to the comparison evaluation in Section 5, we do not apply any filtering to the signals in the following results.

To get a good compromise between time and frequency resolution, we decided to have a FFT window length of 48 s for obtaining the respiration rates in all following parameter examinations. It has the advantage that the 0.17 Hz and 0.25 Hz frequencies from our paced breathing are accurately resolved by the FFT, so they do not suffer from leakage and a simple rectangular window can be used as windowing function. Furthermore, it comprises a frequency resolution of approximately 0.02 Hz and thus the precision of the accuracy measure lies within 1.2 breaths per minute. The window is moved with a step size of one period (6 s at 0.17 Hz and 4 s at 0.25 Hz) over the signal of a certain parameter set, resulting in 7 or 10 distinct windows for the computation of the accuracy. The following sub-sections will list the results from analyzing the data as described in Section 4, using the performance measures as described in Section 4.3.

**Figure 10:**
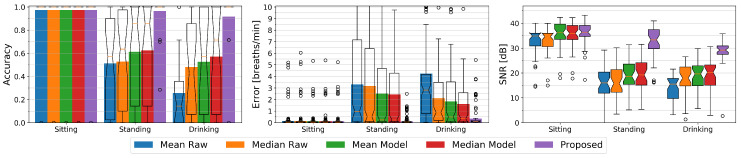
**Left**: The respiration rate accuracies of the chest using a Fast Fourier Transform window with a length of 48 s, for all participants performing three activities (sitting, standing upright, and drinking), for the following methods: Mean Raw, Median Raw, Mean Model, Median Model, and our proposed method. The colored bars show the averages, while overlay box plots show median (middle parts) and whiskers marking data within 1.5 IQR. **Middle**: The error in breaths per minute of the different methods again using bars and overlay box plots. **Right**: The signal to noise ratio (or SNR) of the chest area shows a similar picture to the accuracy performance measures: Sitting results for all methods in a much clearer signal than standing upright, with standing and occlusion (holding a cup, right measures) performing slightly worse than just standing upright.

At the outset of this paper, we argued that related work has primarily looked at solutions where the persons under observation were lying down or sitting still. By introducing a condition where study participants perform activities in different settings including regular self-occlusion gestures while holding a cup and standing in front of the depth sensor, we intend to assess a more realistic activity recognition scenario. We first compare how mean- or median-based methods on the raw depth data would perform, and compare these to how our proposed method performs. To be able to study the effect of the different conditions, we focus on the chest being the most suited body region for respiratory rate detection as for instance justified in [25]. Figure 10 visualizes the accuracy and errors of the sitting, standing, and drinking conditions for all five methods to be evaluated, and gives an insight to the signal quality by providing the respective signal to noise ratios.

For the sitting condition, all methods show an equally high accuracy of 97% and a high signal to noise ratio of about 35 dB to 36 dB. The median SNR values of the raw methods are about 1 dB below those of the model-based methods. This is expected as the model comprises a low-pass filter that reduces the overall noise level. The Raw methods show a mean error of about 0.15 breaths per minute whereas the other methods show average errors of about 0.1 breaths per minute. The differences however are negligible.

Standing introduces slight motion artifacts due to small body movements. Most of the time these movements are unconscious such as for example swaying, but sometimes for example a user relieves a leg or repositions its arms. The mean and median-based methods with or without a model do not have a reference point and can hardly compensate for this additional motion. Their average accuracy as well as their SNR significantly drop to about 52% and 16 dB for Mean Raw and Median Raw, and to about 62% and 19 dB for the Mean Model and Median Model methods as compared to the sitting condition. Their average error similarly increases to about 3.2 breaths per minute for the Raw methods and to about 2.5 breaths per minute for the model-based mean and median methods. Our difference-based method’s accuracy remains stable at 97% with an average error of 0.1 breaths per minute and only the SNR drops a bit to 33 dB.

The drinking condition causes even more body movements and additionally introduces occlusion. The median methods show a higher accuracy and lower error than their respective mean counterparts with 48% vs. 26% and 2.1 vs 4.2 breaths per minute for the raw methods, and 57% vs. 53% and 1.6 vs. 1.8 breaths per minute respectively for the Model methods. As the median typically is more robust against outliers, these methods have a higher chance of not seeing an occlusion or of only suffering from it at a fraction of the time. Due to the occlusion recovery of the model-based methods, these can handle the occlusions better than the raw methods and yield a higher accuracy. Still all these methods cannot compensate for the body movement which remains the main reason of their decreased accuracy. The SNR of the Mean Raw drops to 15 dB while the SNR of the other three methods with 18 dB for the Median Raw and about 20 dB for the other two is slightly higher than for the standing condition. The difference-based method with an average accuracy of 92% and an average error of about 0.34 breaths per minute can handle the occlusion scenario very well although its accuracy is lower than without occlusions. Its SNR also drops a bit to 29 dB, but is still significantly better than that of the other methods.

### The Influence of Respiratory Rate

**Figure 11:**
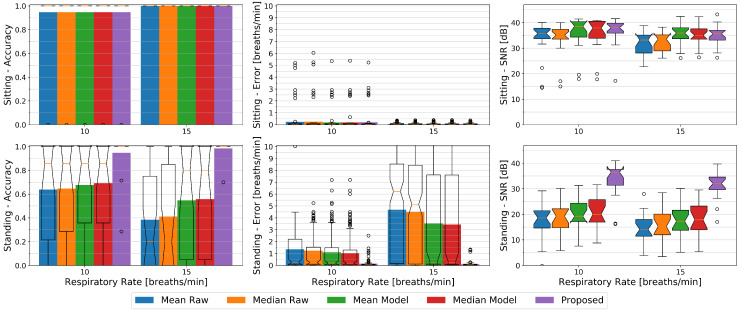
The respiration rate accuracies (**left**), errors (**middle**), and SNR (**right**) of the chest using a Fast Fourier Transform window with a length of 48 s, for the users performing three activities (sitting, standing upright, and drinking) for the following methods: Mean Raw, Median Raw, Mean Model, Median Model, and our proposed method. The colored bars show the averages, while overlay box plots show median (middle parts) and whiskers marking data within 1.5 IQR. Higher rates show a slightly better performance over all methods, especially when non-sedentary.

In Figure 11 we plotted the accuracy, errors, and SNR values of the different methods against the two respiratory rates of 10 breaths per minute (0.17 Hz) and 15 breaths per minute (0.25 Hz) from our paced breathing setup, each divided into the sitting and standing condition.

While sitting, in terms of accuracy the respiratory rate of 15 breaths per minute can with an average of 100% vs. 95% better be observed by all methods and show close to zero average errors in contrast to about 0.2 breaths per minute error rates for the slower respiration. The SNR however is higher at 10 breaths per minute where it lies at about 36 dB for the Raw methods and at about 38 dB for the others. For the faster breathing it lies at about 33 dB for the Raw methods and ranges from 35 dB to 36 dB for the remaining ones respectively.

Except for our proposed method, in terms of accuracy and error rates, all methods behave the other way round for the standing condition where 10 breaths per minutes can better be observed by these methods with an accuracy ranging from 64% to 69% and average errors from 1 to 1.4 breaths per minute. Our proposed method shows an accuracy of 94% for the slower, and 99% for the faster breathing with a mean error of about 0.1 breaths per minute for both. The SNR similarly to the sitting condition drops for the higher respiratory rate a bit for all methods. It lies at about 19 dB for both Raw and the Mean Model methods, at 20 dB for the Median Model, and at 36 dB for our proposed method at 10 breaths per minute. At 15 breaths per minute it ranges from 14 dB to 18 dB in increasing order from Mean Raw to Median Model and has a median value of 32 dB for our proposed method. We argue that the higher respiration frequency interferes stronger with other body movement and thus cannot be detected that easily, but it also is likely that the relatively relaxed low respiration frequency of 0.17 Hz did not introduce as many motion artifacts as the faster one.

## 7. Feature Extraction

In this last experiment, we finally inspect in more detail how the resulting respiration signal out of depth data from our proposed method can be applied for activity recognition. We used for this experiment the dataset from the first experiment, in which 14 participants performed the activities: high-paced meditating, relaxing, and post-exercise recovering. As an additional activity we added reading, in which 11 participants were asked to read aloud the same text across all study participants for several minutes. These are all activities in which all participants were in a standing posture, making it hard to use body posture or body joint sequences to be used to distinguish between these activities. We identified a list of several features that work particularly well to discriminate between these different activities:

- Standard deviation *Std-Spec*, skew *Skew-Spec*, and kurtosis *Kurt-Spec* of frequency spectrum amplitudes.
- Signal to noise ratio *SNR*.
- Median of the time deltas between peaks in the signal *Med-PP*.
- Spectral entropy of the signal obtained in frequency domain *ESP*.
- Standard deviation of the first order time derivative of the signal *Std-Deriv*.

**Figure 12:**
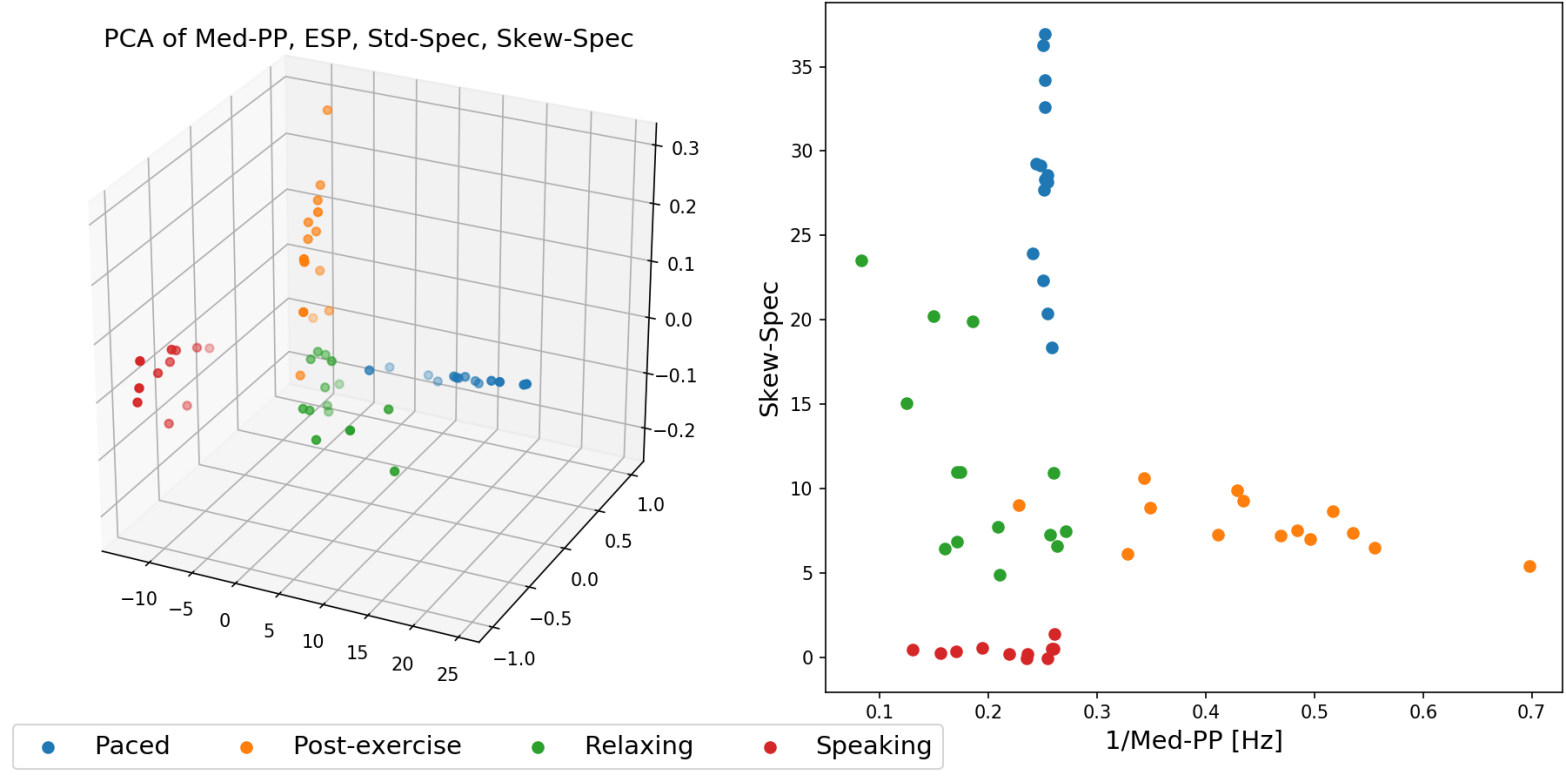
The different activities: paced-breathing meditating, post-exercise recovering, relaxing, and speaking, transformed to feature space. **Left**: The features Med-PP, ESP, STD-Spec, and Skew-Spec reduced by one dimension by a principal component analysis. **Right**: The inverse of the feature Med-PP (to express it in Hz) plotted against Skew-Spec.

Figure 12 shows two feature space plots of the data expressed in feature space of the more promising features. Figure 12, right, highlights that with already two well-chosen features, it would be sufficient to effectively split the data into distinct clusters and that with an ensemble of linear classifiers these four activities could be separated well across the 14 study participants. The one outlier point from the post-exercise activity (in orange) that appears within the relaxing (green) cluster was due to our algorithm failing to provide the correct respiration signal estimation.

## 8. Discussion

In light of the above results, this section will report on these results and discuss the limitations, assumptions and requirements for both our method and the evaluations.

### 8.1. Comparison of Our Method to State-of-the-Art (Wearable or from a Distance)

The majority of current state-of-the-art methods are based on the mean of depth pixels from a certain region of the torso. These methods mostly are either evaluated by visual inspection or by the correlation to a reference signal e.g., from a spirometer and in most cases only comprise a low number of participants. Furthermore, they are evaluated only for lying down or sitting users. This makes a direct comparison difficult. The comparison therefore is performed on extracting the mean from the raw depth frames as well as from low-pass filtered and occlusion recovered versions thereof with improved window alignment as given from our model. Furthermore, we extend the comparison by computing the more outlier-robust median of the same. While sitting all methods perform similarly well with an average accuracy of 97%. In the case of standing, our proposed method outperforms purely mean or median of depth-based methods with an average accuracy of 97% versus about 52% from unprocessed depth data to about 62% with low-pass filtered data and improved window alignment from our model. When it comes to the drinking activity with regular self-occlusions happening, the purely mean-based method’s accuracy drops to 26% while our proposed method’s accuracy still maintains an average accuracy of about 92% and the median and 1.5 IQR accuracies being at 100%. The median and model-based methods are more robust than the mean but can with average accuracies of about 48 to 57% not compete with our proposed method. In summary it has been shown that our difference-based method is superior to these in the case of standing and self-occlusions and has similar performance while sitting (also see Section 6).

For PCA-based methods, according to the related work, the users are required to keep still, e.g., to lie in supine position, and to wear very tight clothing with no folds. To build the PCA model, depending on the respiratory rate, about 100 frames are required from exactly the same bounding window that solely contains breathing movements. We therefore argue that in more realistic scenarios movement and surface deformations will dominate the principal components, making it almost impossible to select breathing related components. Even with a valid model, motion artifacts or occlusions will hinder a correct estimation of respiration. We therefore consider state-of-the-art PCA-based methods as not being applicable to our dataset or for use in scenarios with many motion artifacts due to a moving body in general.

Volume-based methods compute the change of the torso volume from a triangulated body region that is given by the shoulder and hip joints. In case of only a front facing depth camera, no reliable data is available that bounds the volume to the back. In the related work, it often remains unclear whether the volume spans up to a certain constant threshold or is computed from the edges of the surface patch. Using the edges of the torso or a defined torso area is not a reliable process as it is subject to unreliable bounding window alignments, movements, surface deformations, and additionally is strongly correlated with breathing movements as we found in our experiments (see Figure 5). Also, the sides of a torso are only visible to a certain extent and are regularly occluded by the arms. In papers that do report on the volume bounding, the volume was bound by a constant threshold in the distance and thus increases or decreases with motion. For related work that does not explicitly report on the bounding, we assume the same. The volume in either way is a weighted sum of depth measures, each subtracted by a constant as given by the fixed boundary at the back. Weighting happens according to the relative surface area covered by the individual depth pixels. The volume can well be approximated by dividing the sum of all depth measures by the number of depth measurements and multiplying the result with the extents of the observed surface region. The volume therefore basically is a weighted average, so we consider it as being a mean-based method with additional computational overhead that moreover may also be less accurate as already shown in [30].

Wi-Fi-based respiratory rate detection is a promising method as it comes at almost no cost, given a Wi-Fi enabled infrastructure. According to the related work, the Wi-Fi antennas however usually require a particular setup and alignment towards the user [21]. The antennas in most cases are in front of a lying, sitting, or standing still person within the sensing range of about 1 to 3 m and, according to [22], respiration sensing tends to fail when the observed person performs hand gestures. We assume that even small body movements while having to stay still for a while, as observed in our experiments, will cause noticeable signal distortions. If Wi-Fi-based respiration monitoring works in a realistic environment, i.e., sender and receiver not in the same room or not within a range of a few meters and the observed person being randomly aligned, and how well this method performs if not remains to the best of our knowledge an unsolved research question. Furthermore, if two or more users are within the detection range, it remains unclear how to assign a respiration signal to the correct person or how to distinguish different breathing signals that are likely to interfere on the common carrier, especially when they overlap in frequency domain. A depth-based method such as ours has the benefit that users can be further away, can be distinguished and assigned the correct respiration signal that only is present on depth pixels covering a user’s torso, and that changing environmental conditions, such as other persons walking through in the background of the scene, have less of an effect. Also, depth images provide a valuable insight in user movements that can be exploited to reduce motion artifacts. The fact that the depth camera can easily be located and blocked is both an advantage (transparency to users) and a disadvantage of our method.

While special devices, ranging from spirometers to respiration belts, in general yield optimal results, they tend to be expensive and uncomfortable to wear for longer stretches of time. Furthermore, they have to be made available to the user and may need a supervisor for the setup. Having to wear a mask or respiration belt that might need occasional readjusting (due to it being too tight or too loose) from time to time certainly imposes distraction to the user. Other, less distracting wearable devices such as PPG equipped smart watches or fitness bands also have to be available to the user and need to be connected to the monitoring system. In applications where users for a particular activity recognition application are not willing or not able to wear on-body sensors, a depth-based method has been found a viable alternative.

### 8.2. Limitations of Our Dataset and Method

Our dataset only comprises use cases where we assume that, while performing an activity, a user generally faces the depth camera while in a sedentary or standing position. This includes small swaying motions, repositioning movements to either side, e.g., when switching from one leg to another, small body rotations, and occlusion events (such as arm gestures) at different distances and respiratory rates. A more general approach should also consider different static or dynamic body alignments towards the depth camera, especially leaning forward or rotating to either side as well as fast body movements and motion towards the camera. Another important aspect to be investigated is the effect of clothing. Our participants are wearing a big variety of indoor clothing, but our dataset yet lacks a systematic classification of the clothing styles. For such a more general application, where users can be walking, running (on a treadmill), or performing fast body movements, our method yet has to be validated and will likely perform poorly.

Our proposed method relies heavily on tracking the torso and the differences between a region of the torso that is less affected by respiration at the throat, and a heavily respiration-affected area within the torso. As the throat’s region is relatively small, it is susceptible to noise, occlusions, and clothing effects such as a moving collar. This makes our method more susceptible in winter or colder climates, for instance, when users might wear heavier attire that completely covers the throat region, too. Another limitation is that our model cannot deal with large rotational offsets and, at the moment, requires the user to keep a steady distance to the depth camera. The latter can be solved by rescaling the input frames or the model, large rotations on the other hand are more challenging. In a similar implementation, they would for instance require a three dimensional model and an iterative closest point algorithm to match the input frames to the body surface, making it hard to keep real-time performance especially on embedded platforms. Small rotational or spatial movements however are present throughout our whole dataset, especially during the standing postures. Our model can adapt to these and, due to the difference-based signal extraction, our method performs remarkably well under these conditions.

Another issue is that in some cases low frequency components dominate in the frequency domain, even when a clear respiration signal is present in the time series. An alternative would be to use peak detection or a zero crossings method to find the respiratory rate more timely and without the effect of lower frequencies showing up. These methods on the other hand have to deal with noise that especially at higher distances significantly increases and may have decreased precision due to false positives or signal distortions. Different types of environmental noise can thus impact the quality of our method’s resulting respiration signal. It is possible that adaptive digital filtering noise cancellation techniques be integrated to provide more accurate results in future versions of our proposed method.

Since the depth imaging frames can contain a user anywhere in the frame, we expect our method to work well with multiple users, as long as these do not block each other. This likely becomes challenging for detecting users’ positions and joints in the depth frames accurately as more users are present. We did not pursue this multi-user scenario further, as activity recognition applications that detect and interact with multiple users simultaneously are less common.

**Figure 13:**
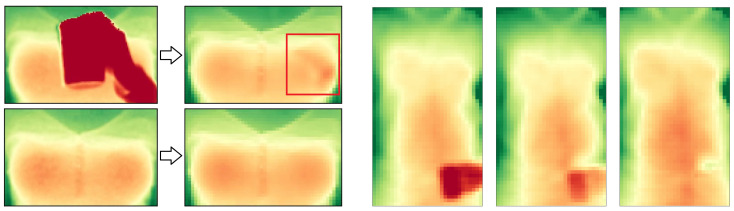
**Left**: Comparison of occlusion vs no occlusion on the prediction. Note the difference in the highlighted area. **Right**: Example of the adaptiveness of the model: An initial occlusion fades away as soon as the occluded area gets visible.

Finally, we saw that another critical component of our method, the surface reconstruction, does not always deliver perfect results, as for instance illustrated in Figure 13 (left). It however does ensure that over time the recovered parts are elevated accordingly to the surrounding pixels while keeping sufficient surface detail to distinguish between occluded and non-occluded regions in the following input frames. Moreover the model is adaptive to small surface deformations and does not require a valid initialization. Any occlusions erroneously incorporated into our model will fade away as soon as the occluding object moves away, leaving behind a better surface approximation as depicted in Figure 13 (right), but this does take several frames.

## 9. Conclusions and Future Work

In this article, we presented an approach that monitors the respiration from users facing a depth camera, which is particularly useful in activity recognition scenarios: Our approach focuses on robustly segmenting the data from the user’s torso in the depth images, using the detected user’s body joints that are part of many optical activity recognition approaches already, and modeling this torso area over time. We showed that it is possible to detect the breathing rate, even when the person in question is standing upright and occasionally occluding their torso. Three sets of experiments were performed collecting and analyzing data from 24 study participants, comparing our approach with a commercial wearable respiration monitor, examining crucial activity-related parameters such as different respiration rates and different activities (such as sitting, standing, and standing while performing drinking gestures), and which respiration-based features can be used in particular to distinguish between activities.

This paper’s experiments can be summarized in the following four findings:

- Estimation of respiration rate from depth data becomes significantly harder when the observed person is standing freely. When, as in prior work, persons are lying down in a supine position or sitting in a chair, body movement is restricted significantly. Our studies showed that even when persons are standing in front of a depth camera, traditional optical respiration estimation approaches tend to fail when (1) the body sways through motion from hips and legs, as well as (2) occlusions, in particular those coming from the persons gesturing themselves. Previous methods show here significant drops (to around 26%) in accuracy.
- The observed torso region plays a crucial role in the reliability of the respiratory rate detection. Especially in the standing or occlusion scenarios, our results confirm that the chest region is highly recommended for capturing the respiration signal, whereas observing the stomach area performs less well. If user movements and occlusions are not too large, our method’s torso tracking and filtering performs well and reaches a comparable performance to commercial respiration monitors.
- The respiration signal that our approach provides as an estimate of users’ breathing can be used to distinguish between particular activities. In an analysis of several possible descriptors for the breathing signal, especially the features Med-PP, ESP, STD-Spec, and Skew-Spec were found to be promising to be used in activity recognition.
- Person-dependent features, such as clothing, have been found to greatly influence what regions are primarily important to derive the respiration signal. The method can therefore be improved by not resorting to predefined areas on chest or abdomen, but instead using an adaptive method considering multiple regions. Performance results between participants under the same conditions were surprisingly variable.

Our ongoing work is exploiting the fact that our method is fairly light-weight, requiring only limited memory and moderate processing resources, and can be implemented on an embedded system close to the depth sensor. This is especially interesting for more longitudinal deployments where persons’ activities and breathing rates are to be estimated locally and in real time, for instance to enable direct feedback during relaxation exercises or in fitness training applications.

This paper’s anonymized dataset with depth data and respective body joints locations, as well as our method’s source code and the python experiment scripts that were used for validating our proposed method are available to support the reproduction of our method and results, and can be obtained by contacting the first paper author or visiting https://ubicomp.eti.uni-siegen.de/home/datasets/.

All subjects gave their informed consent for inclusion before they participated in the study. The study was conducted in accordance with the Declaration of Helsinki, and the protocol was approved by the Ethics Committee of the University of Siegen (ER_12_2019).
